# Reversal of diabetes by an oral *Salmonella-*based vaccine in acute and progressive diabetes in NOD mice

**DOI:** 10.1371/journal.pone.0303863

**Published:** 2024-05-23

**Authors:** Jacob Cobb, Jeffrey Rawson, Nelson Gonzalez, Chris Orr, Fouad Kandeel, Mohamed I. Husseiny

**Affiliations:** Department of Translational Research & Cellular Therapeutics, Arthur Riggs Diabetes & Metabolism Research Institute, Beckman Research Institute, City of Hope National Medical Center, Duarte, California, United States of America; Universitat Ramon Llull, SPAIN

## Abstract

Type 1 diabetes (T1D)-associated hyperglycemia develops, in part, from loss of insulin-secreting beta cells. The degree of glycemic dysregulation and the age at onset of disease can serve as indicators of the aggressiveness of the disease. Tracking blood glucose levels in prediabetic mice may demonstrate the onset of diabetes and, along with animal age, also presage disease severity. In this study, an analysis of blood glucose levels obtained from female NOD mice starting at 4 weeks until diabetes onset was undertaken. New onset diabetic mice were orally vaccinated with a *Salmonell*a-based vaccine towards T1D-associated preproinsulin combined with TGFβ and IL10 along with anti-CD3 antibody. Blood glucose levels were obtained before and after development of disease and vaccination. Animals were classified as acute disease if hyperglycemia was confirmed at a young age, while other animals were classified as progressive disease. The effectiveness of the oral T1D vaccine was greater in mice with progressive disease that had less glucose excursion compared to acute disease mice. Overall, the *Salmonella*-based vaccine reversed disease in 60% of the diabetic mice due, in part, to lessening of islet inflammation, improving residual beta cell health, and promoting tolerance. In summary, the age of disease onset and severity of glucose dysregulation in NOD mice predicted response to vaccine therapy. This suggests a similar disease categorization in the clinic may predict therapeutic response.

## Introduction

Type 1 diabetes (T1D) is characterized by autoimmune-mediated killing of insulin-producing pancreatic beta cells [1,2]. The onset of metabolic dysregulation in T1D occurs after loss of 60% to 80% of the total beta cell mass [3,4]. Beta cell loss can occur suddenly or gradually in a relapsing-remitting fashion [5,6]. However, real-time direct assessment of the islet microenvironment in individuals with T1D is still not possible [7,8]. The rodent version of T1D that transpires in NOD mice with aging provides a means to study rapid versus delayed onset T1D. Impaired glucose metabolism was suggested as a marker for diabetes progression in prediabetic female NOD mice at different ages [9,10]. While not the same in some regards to clinical disease, NOD mice also provide a means of testing T1D therapies [11–13]. Disease heterogeneity, demonstrated by the severity of beta-cell loss or the aggressiveness of beta-cell autoimmunity likely accounts for ineffectiveness of therapy [10,14].

In this study, we analyzed our data to determine if the age of disease onset and severity of diabetes predicted therapeutic efficacy of an oral *Salmonella*-based vaccine to reverse diabetes [15–20].

## Materials and methods

### Mice

Female NOD/ShiLtJ (NOD) mice (Jackson Laboratories) were maintained under pathogen-free conditions. Animals received high quality care consistent with the Public Health Service Policy. The animal care facility at City of Hope National Medical Center is fully accredited by the Association for Assessment and Accreditation of Laboratory Animal Care International (AAALAC). All procedures were conducted in accordance with the Declaration of Helsinki and approved by the Institutional Animal Care and Use Committee (IACUC# 18017). Mice were euthanized by cervical dislocation under anesthesia using inhalation of 5% isoflurane. During the study animals were observed for visual clinical signs such as loss of body weight, dehydration, hunched posture, poor coat quality, and grimace will be used to assess overt signs of pain. All efforts were made to minimize animal discomfort.

### Blood glucose measurement

Blood glucose levels were measured twice a week starting at 8 weeks of age using a One Touch Ultra glucometer (LifeScan, Milpitas, CA). Mice were considered to have disease onset when blood glucose values ≥200 mg/dL for two consecutive measurements [21–23]. For this analysis, acute onset of T1D was characterized as blood glucose < 200 mg/dL prior to full onset [24]. Progressive onset of disease was characterized as at least one blood glucose reading ≥ 200 mg/dL prior to full disease onset [24].

### Animal experiments

The attenuated strain of *S*. *typhimurium* was employed for oral vaccination as we described [15–17]. Diabetic NOD mice were vaccinated via oral gavage with *Salmonella* expressing autoantigen preproinsulin and immunomodulators (TGFβ and IL10) on days 0 and 7. Vaccinated mice were given anti-CD3 mAb (2.5 μg./mouse) via intraperitoneal injection for 5 consecutive days starting one day before vaccination start [16–20,25]. Blood glucose values were monitored biweekly for 14 weeks (100 days) after vaccination. Disease reversal was defined as a blood glucose level consistently < 200 mg/dL.

### Histological evaluation

Hematoxylin and eosin (H&E) staining was performed on formalin-fixed pancreatic paraffin sections. The islets were observed under light microscopy at 20× or 40×, enumerated and graded in a blinded fashion for islet infiltration [15,16] from mice with acute (n = 11) and progressive (n = 23) onset of diabetes. Four slides from each mouse at different section levels were scored.

### Immunostaining

Immunostaining was performed on formalin-fixed pancreatic tissue sections for insulin using guinea pig anti-insulin polyclonal antibody (Dako) and donkey anti-guinea pig secondary antibody conjugated with FITC (Jackson ImmunoResearch). Parallel sections were quantified based on DAPI staining for nuclei (blue) and insulin (green). Fluorescent images were acquired on a ZEISS inverted LSM700 microscope, using ZEN-lite digital imaging software (Carl Zeiss, Oberkochen, Germany) for processing. The fraction of beta cells represents the percentage of insulin positive cells in relation to the total number of cells per islet. The islets were from pancreata of mice with acute (n = 11) and progressive (n = 12) onset of diabetes and counted from 4 slides from each mouse at different section levels.

### Cytokine measurement

Soluble circulating cytokines and chemokines in serum samples from mice were quantified using a MILLIPLEX MAP Mouse High Sensitivity T Cell Premixed Panel—Immunology Multiplex Assay (Millipore) and a Bio-Plex analyzer (Bio-Rad, Hercules, CA) [19].

### Statistical analyses

To account for differences in variance between groups, the unpaired Welch’s *t* test was used to analyze the differences between acute and progressive onset of disease, The Chi-square test was used to compare acute or progressive onset associated with disease reversal or lack of reversal. A p < 0.05 was considered significant. Two-way ANOVA was used for analysis of the degree of immune cell infiltration in islets. Statistical analysis was performed using GraphPad Prism 10 software.

## Results

### Age distribution at disease onset in NOD mice

**Fig 1:**
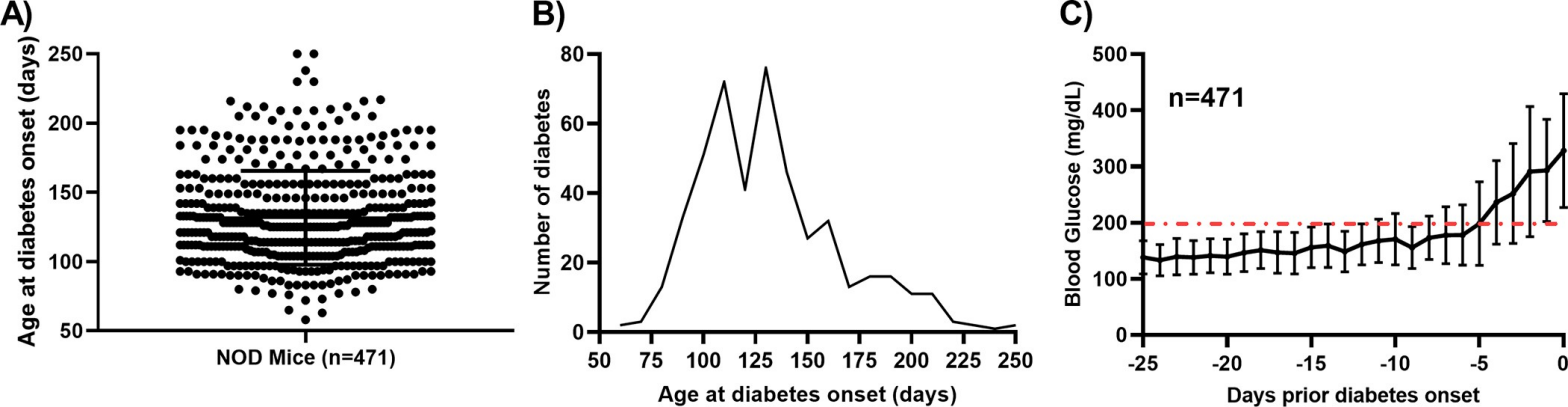
Natural history of blood glucose levels in a large number of female NOD mice. (A) The age at diabetes onset varied considerably, occurring as early as 60 and as late as 245 days of age. (B) Distribution of diabetes at different ages. (**C**) Blood glucose levels in NOD mice (n = 471) were recorded 2 times per week starting at 8 weeks of age. Blood glucose levels are synchronized for diabetes onset (set to day 0) defined as two consecutive glucose readings ≥200 mg/dL. Data are presented as mean ± SD. The data here includes only the 471 mice that developed diabetes.

NOD mice (n = 600) had blood glucose levels monitored from 50 to 250 days of age. We found that the earliest age of disease onset was 58 days (9 weeks) (Fig 1A), with a peak onset at 19 weeks (Fig 1B). The incidence of diabetes was 55% (327 of 600) by 140 days (20 weeks), 68% (409 of 600) by 175 days (25 weeks), 77% (460 of 600) by 210 day (30 weeks), and 79% (471 of 600) by 245 days (35 weeks) (Fig 1A).

### NOD mice show a progressive increase in blood glucose levels weeks prior to diabetes onset

Analysis of the blood glucose levels taken over 24 days prior to diabetes onset was done (Fig 1C). Interestingly, blood glucose levels averaged less than 200 mg/dL from 7 to 24 days prior to disease onset. However, within one week of disease onset, as a collective, blood glucose levels increased, with the most rapid rise within 5 days of overt disease (Fig 1C).

### Progressive versus acute onset disease based on blood glucose values

**Fig 2:**
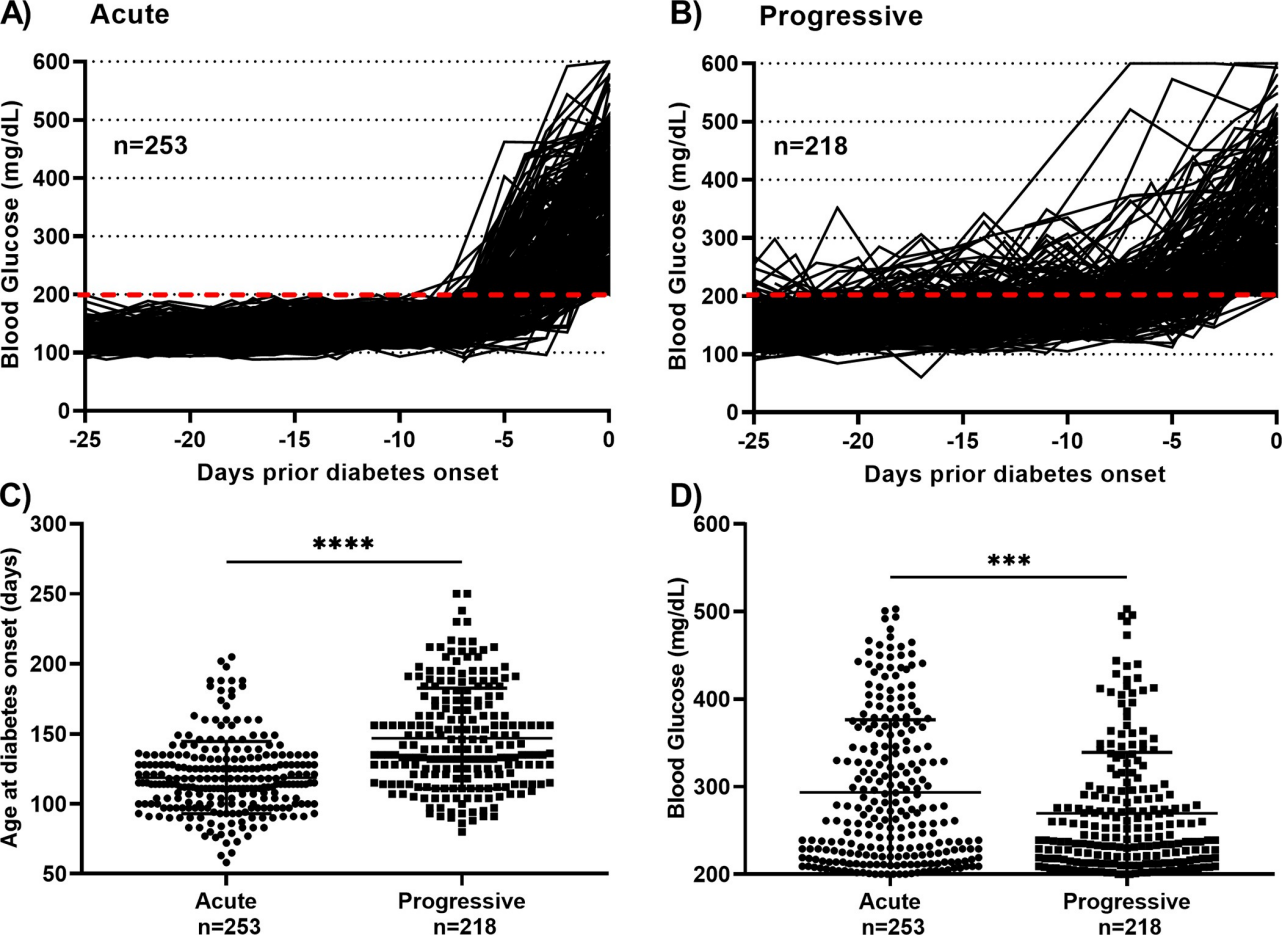
Acute or progressive onset of diabetes in female NOD mice. Data displayed as ante meridiem blood glucose levels in female NOD mice for the 25 days prior to diabetes onset (set to day 0). (A) Acute onset (n = 253) was characterized as blood glucose < 200 mg/dL prior to onset, while (B) progressive (n = 218) was characterized as at least one blood glucose level ≥ 200 mg/dL prior to onset. (C) Mice belonging to the acute-onset group were significantly younger at the time of disease onset compared with mice with progressive disease onset (**** p < 0.0001, Welch’s *t* test). (D) Blood glucose levels were significantly increased in acute onset compared to progressive onset in NOD mice on the day of diagnosis (***p < 0.001, Welch’s *t* test). Data are presented as mean ± SD.

Prior to diabetes onset, considerable variability in blood glucose values with wide standard deviation at each time point was noted (Fig 1C). To distinguish between animals that maintained a constant blood sugar level up to the acute onset of disease from those animals that displayed variable blood sugar up to a progressive onset, we divided mice into two groups based on blood glucose fluctuations in the 25 days prior to the onset. In fact, 253 of 471 of the mice (54%) had blood glucose levels consistently below 200 mg/dL which was followed by sudden onset of diabetes, that is acute disease (Fig 2A). The remaining mice (218 of 471, 46%) had at least one blood glucose > 200 mg/dL prior to progressive disease onset (Fig 2B).

Animals with acute onset of diabetes were significantly younger at diagnosis than those with progressive disease (119 versus 147 days, p < 0.0001) (Fig 2C). Animals with acute onset of T1D were significantly more hyperglycemic at diagnosis than those with progressive disease (294 versus 270 mg/dL, p = 0.0007) (Fig 2D).

### A *Salmonella*-based vaccine reverses diabetes more effectively in mice with progressive disease onset

**Fig 3:**
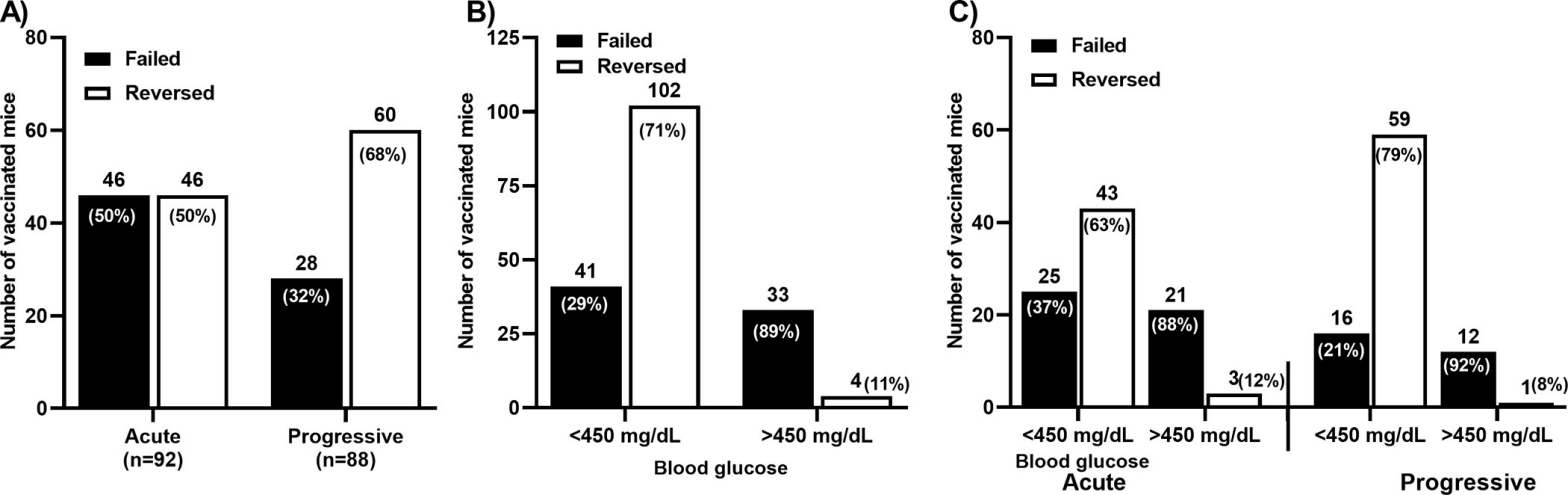
A *Salmonella*-based vaccine reverses diabetes in NOD mice with acute and progressive onset of diabetes. Diabetic NOD mice were vaccinated orally at days 0 and 7 after diagnosis. Mice also received anti-CD3 mAb for 5 consecutive days starting one day prior vaccination start. The numbers and percentages of each group are indicated. (A) Reversal rate of diabetes in acute versus progressive onset of disease in mice after vaccination. (B) Reversal rate of diabetes in vaccinated mice with blood glucose < 400 mg/dL or > 400mg/dL. (C) Reversal rate of diabetes in acute versus progressive disease onset and as related to the starting blood glucose values at the time of vaccination.

It was not known if the S*almonella*-based vaccine we developed was equally effective regardless of disease phenotype. To begin to assess this, we interrogated the effects of the vaccine in relation to acute or progressive disease. After mice were confirmed to be diabetic, a subgroup (n = 180) of mice were given orally a *Salmonella*-based vaccine and blood glucose values were monitored. Overall, 106 out of 180 vaccinated mice (59%) experienced reversal of diabetes while 74 (41%) did not respond. This subset analysis agreed with trends in half of the cohort with 49% (n = 88) of mice exhibiting progressive onset, and the remaining half 51% (n = 92) exhibiting acute onset of diabetes (Fig 3A). The diabetes reversal rate was lower in mice with acute disease (50%, n = 46) than in mice with progressive disease (68%, n = 60) (Chi-square = 6.142, p = 0.013) (Fig 3A).

Extending this, animals were classified into those with blood glucose < 400 and those with blood glucose > 400 mg/dL **(**Fig 3B**)**. As expected, the efficacy for disease reversal was highest in those animals with lower starting blood glucose values. The reversal rate (71%, n = 102) was higher in animals with starting blood glucose levels < 400 mg/dL (Fig 3B) and only 29% (n = 41) (Chi-square = 44.47, p < 0.0001) of such animals failed to reverse. Animals with starting blood glucose level > 400mg/dL were reversed at a lower rate (11%, n = 4) with 89% (n = 33) (Chi-square = 44.47, p < 0.0001) of the animal not reversing (Fig 3B).

The reversal rate (79%, n = 59) (Chi-square = 25.73, p < 0.0001) was higher in animals with progressive diabetes onset and blood glucose levels < 400 mg/dL (Fig 3C). However, 92% (n = 12) (Chi-square = 25.73, p < 0.0001) of the animal with progressive diabetes onset and blood glucose levels > 400 mg/dL failed to improve after vaccination. Animals with acute onset and blood glucose level < 400mg/dL responded to the vaccine in a majority of cases (63%, n = 43) compared with 37% (n = 25) (Chi-square = 18.26, p < 0.0001) mice that did not (Fig 3C). These data demonstrate that vaccine effectiveness was positively associated with progressive disease onset and lower blood glucose.

### A *Salmonella*-based vaccine preserves beta cells and reduces insulitis in mice with progressive onset of diabetes

**Fig 4:**
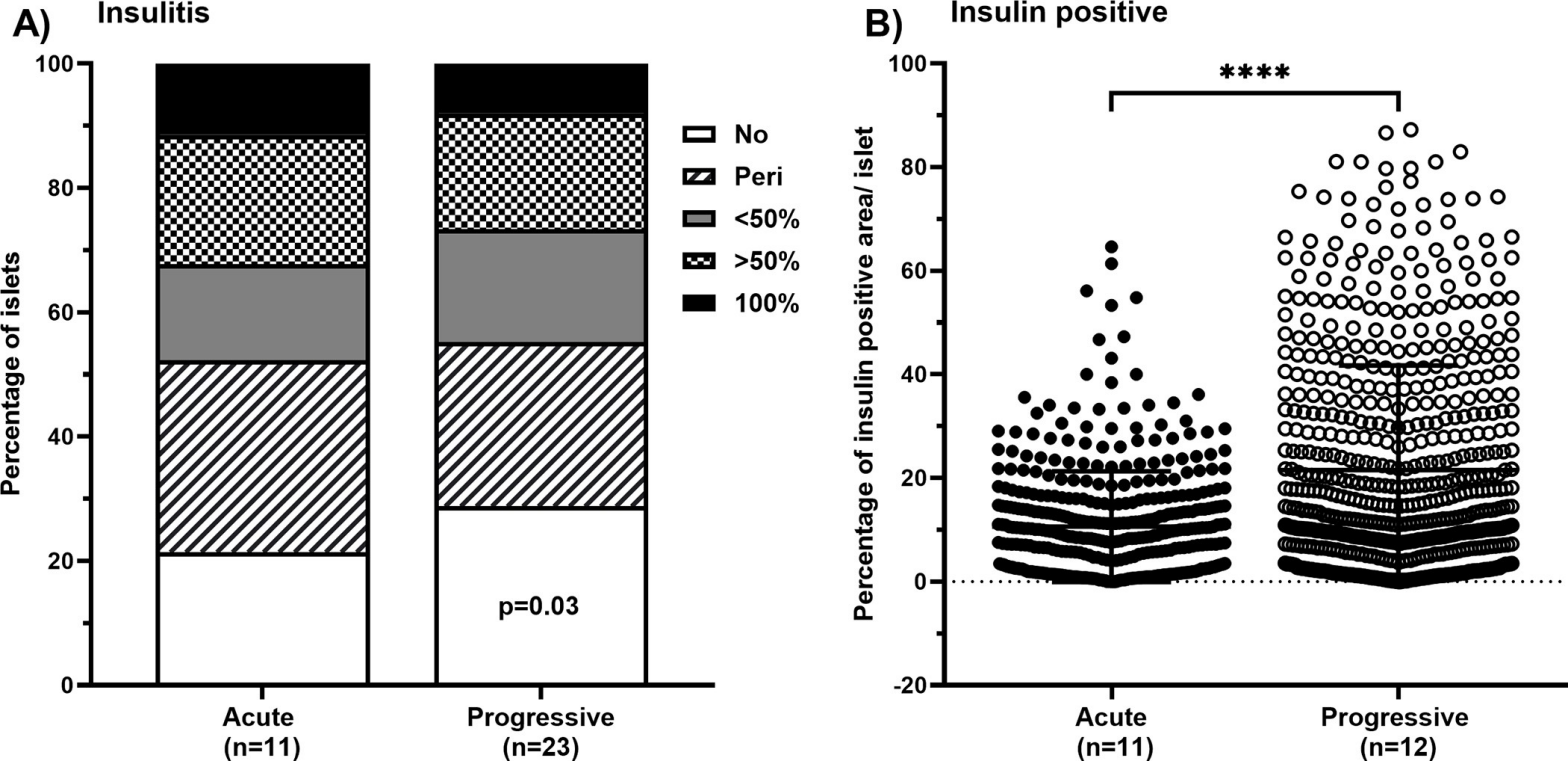
A *Salmonella*-based vaccine is more effective at reducing immune cell infiltration and preserving insulin-positive cells in islets in mice with progressive onset of diabetes. (A) Pancreatic paraffin sections from vaccinated mice were stained with H&E. Islets were observed under light microscopy, enumerated, and graded in a blinded fashion for islet infiltration. The statistical significance was calculated with two-way ANOVA and significance level indicated (* p < 0.05). (B) Pancreatic paraffin sections were immune stained for insulin and the fraction of beta cells was quantified in each islet and shown as the percentage of insulin positive cells per islet. The data is presented as the mean ± SD. The statistical significance was calculated using Welch’s t test for unpaired values and the significance indicated by asterisks (**** p < 0.0001).

T-cell infiltration into pancreatic islets is a key component of autoimmune diabetes. Lymphocyte infiltration in pancreatic tissue sections from vaccinated mice with progressive onset were scored and results compared to pancreatic tissue sections from vaccinated mice with acute onset of diabetes (Fig 4A). Administration of a *Salmonella*-based vaccine reduced lymphocyte counts and protected islets from inflammation in mice with progressive (two-way ANOVA, p = 0.03) compared with the acute onset of diabetes **(**Fig 4A**)**.

The percentage of insulin-positive cells (beta cell area) for each islet was also quantified in the pancreas sections stained for insulin from vaccinated mice with progressive and compared with the acute onset of diabetes (Fig 4B). Islets from vaccinated mice with progressive disease displayed significantly higher percentages of insulin positive cells compared to islets from vaccinated mice with acute onset of diabetes (Fig 4B, Wilch’s t test, p < 0.0001).

### A *Salmonella-*based vaccine did not substantially alter serum cytokines in mice with progressive and acute onset of diabetes

**Fig 5:**
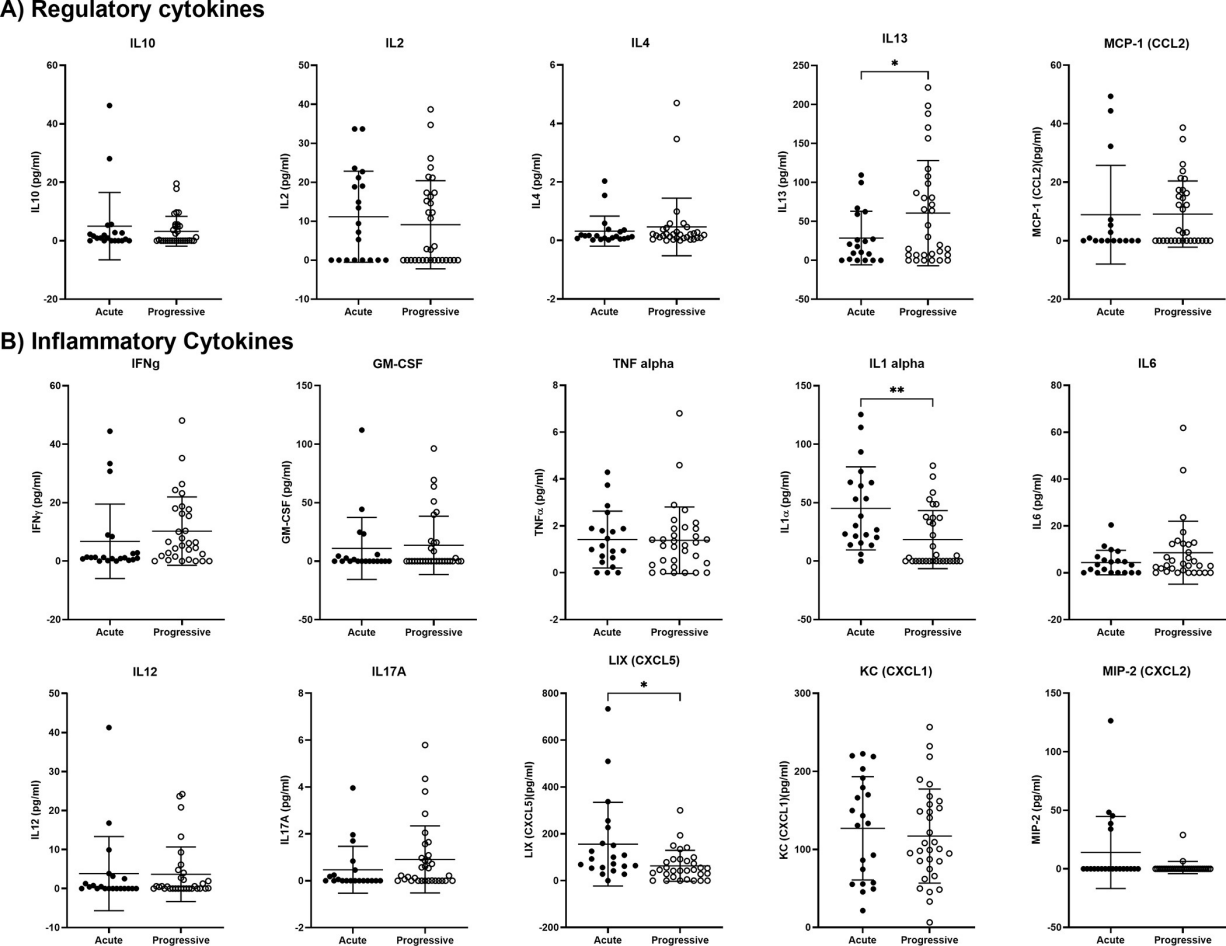
Cytokines profiles of vaccinated mice with progressive and acute onset of diabetes. Serum was collected from vaccinated mice and cytokines were quantified. (A) Levels of regulatory cytokines IL10, IL2, IL4, IL13, and CCL2. (B) Levels of pro-inflammatory cytokines IFNℽ, GM-CSF, TNFα, IL1α, IL6, IL12, IL17, CXCL5, CXCL1, and CXCL2. Data presented as means ± SD from vaccinated mice with acute (n = 21) and progressive (n = 32) onset of diabetes. Statistical significance between acute and progressive onset was determined by the Welch’s t test for unpaired values (* p < 0.05, and ** p < 0.01).

To assess if the vaccine induced tolerance, circulating tolerogenic and inflammatory cytokines were quantified. Luminex beads were used to measure cytokines in the serum of vaccine treated mice (Fig 5). Vaccine treatment was associated with significantly higher levels of the regulatory cytokine IL13 in mice with progressive compared with the acute onset diabetes (Fig 5A, Wilch’s t test, p = 0.03). The levels of circulating IL10, IL2, IL4, and chemokine CCL2 remained unchanged in vaccinated mice with acute and progressive disease onset (Fig 5A). Also, vaccine-treated mice with progressive disease onset had decreased levels of the inflammatory cytokines IL1α and chemokine CXCL5 compared with the mice with acute disease onset (Fig 5B, Wilch’s t test, p = 0.005, p = 0.03). No changes were noted in the circulating levels of proinflammatory cytokines (IFNγ, GM-CSF, TNFα, IL6, IL12, and IL17), and chemokines CXCL1, and CXCL2 regardless of the rapidity of disease onset (Fig 5B).

## Discussion

In T1D, the effectiveness of immune therapy is influenced by the degree of glycemic dysregulation and the age at disease onset [14,26]. We conducted an analysis of a large historical cohort of female NOD mice as they transitioned into T1D and after and related this to post-vaccination outcomes. Some 60−90% of female NOD mice develop diabetes with onset occurring between the ages of 12 and 30 weeks [27–31]. Herein, the earliest time of disease onset was 10 weeks of age, with a peak onset at 19 weeks. By 30 weeks of age, 77% of the mice were diabetic. Overall, the incidence of diabetes in female NOD mice in our study was somewhat less than reported [27–30]. This may be due to presence of *Salmonella* or other environmental factors [29,32]. Other studies showed that by 30 weeks of age, 72% of female and 39% of male NOD mice developed diabetes [33].

A longitudinal assessment of blood glucose levels in NOD mice demonstrated two patterns of prediabetic disease onset, acute and progressive. In relation to these disease patterns, the effectiveness of an oral diabetes vaccine was assessed. Acute onset of diabetes was more common in younger animals whereas older mice were more likely to display progressive disease onset [24]. This is similar to clinical observations wherein acute onset disease is more common in young individuals, while those diagnosed with T1D as adults experience a gradual impairment of blood glucose regulation [34,35]. Similar patterns were noted in our murine studies. Not surprisingly, mice with progressive disease onset responded more favorably to the oral vaccine than those with acute disease onset. Furthermore, we found that the initial blood glucose value at the time of vaccination was an important predictor of therapeutic efficacy. The majority of diabetic NOD mice with initial blood glucose levels < 400 mg/dL demonstrated normal blood levels after vaccination. Interestingly, the vaccine promoted tolerance in all vaccinated mice regardless the onset of the disease. However, regulatory cytokine IL13 was higher and pro-inflammatory IL1α and CXCL5 lower in older vaccinated (progressive) than younger (acute) mice. Additionally, vaccinated progressive disease mice had less islet inflammation and more beta cells versus vaccinated acute disease onset mice. This suggests that the vaccine reversed diabetes when given after disease onset and that new onset disease is associated with residual beta cell function in some instances [25]. These data suggest that consideration of disease onset may help to inform therapeutic trials in clinical T1D.

The timing of therapy is crucial and influenced by the disease process. For example, the levels of C-peptide, as a marker of beta cell function, decreased more slowly in individuals that were older at diagnosis [36]. On the other hand, the loss of beta cells in NOD mice occurs quickly [37] although the impact of aging on this loss has not been investigated. The reasons behind the variation in the rate of disease progression remain an area of inquiry but are likely related to immunological or beta cell differences. For example, immunological repertoire diversity is diminished in elderly individuals or the production of relevant antigens or the stress tolerance of beta cells may differ as a function of age. It is fair to suggest that the design of clinical trials take this into consideration [28].

Since there is no exact marker of disease progression that can be used to compare humans and mice, it might be difficult to determine when to start therapy for T1D. The number of antigen-specific T-cells in mice or autoreactive T-cells and autoantibodies in humans may indicate the point in disease progression at which the efficacy of therapy will be greatest and may help to determine when to initiate therapy [28]. A greater understanding of the correlation between intervention and clinical outcomes could be obtained through a direct measurement of beta-cell death such as measuring circulating levels of unmethylated insulin DNA as a marker of impending beta-cell death [38,39].

The findings of this study should be tempered by several caveats. First, the cohorts of mice that comprised the final data set were from studies conducted at different times. Thus, batch effects may have been at play. Second, the age and blood glucose level at which NOD mice develop diabetes varies. Third, NOD mice are much less genetically varied than individuals with clinical T1D. This homogeneity in the model likely contributes to the success of therapy in mice as compared to individual with clinical T1D [40]. And, the incidence of spontaneous diabetes in the NOD mice is ~60–90% in females and much less in males [29,41–44]. Thus, only female NOD mice are traditionally studied. Thus, these data may not obtain in male mice.

In summary, the results herein may have clinical implications for identifying individuals more likely to respond to therapy. We speculate that *Salmonella* vaccine will be more effective in individuals with progressive disease. The categorization of acute versus progressive disease onset should be considered when designing preclinical and clinical trials, interpreting data from the same, and translating study findings into practice.

## Supporting information

S1 File (XLSX)
